# Impact of Parental Narcissistic Personality Disorder on Parent-Child Relationship Quality and Child Well-Being: A Systematic Review

**DOI:** 10.7759/cureus.100229

**Published:** 2025-12-27

**Authors:** Eirini Orovou, Vaidas Jotautis, Eleni Vousoura, Ioannis Koutelekos, Nikolaos Rigas, Antigoni Sarantaki

**Affiliations:** 1 Midwifery, University of Western Macedonia, Ptolemaida, GRC; 2 Medicine, Kauno Kolegija Higher Education Institution, Kaunas, LTU; 3 Psychology, National and Kapodistrian University of Athens, Athens, GRC; 4 Nursing, University of West Attica, Athens, GRC; 5 Midwifery, University of West Attica, Athens, GRC

**Keywords:** child and youth mental health, child attachment, child well-being, grandiose narcissism, mental health outcomes, narcissistic effect, narcissistic personality disorder, parent–child relationship, parenting, vulnerable narcissism

## Abstract

Parental narcissistic personality disorder (NPD) and narcissistic traits have been increasingly implicated in maladaptive parenting behaviors, emotional unavailability, and disrupted parent-child relationships, all of which are critical determinants of children’s psychological and relational development. Despite growing clinical concern, empirical findings on the impact of parental narcissism on child mental health and attachment-related outcomes remain fragmented and conceptually inconsistent. This review synthesizes quantitative research published between 2015 and 2024 to examine the effects of parental NPD and narcissistic traits on parent-child relationship quality and child psychological outcomes. A systematic search of major electronic databases identified eight eligible empirical studies. Across studies, parental narcissism was associated with poorer relational and psychological outcomes in children, with effects varying by narcissism subtype, trait facet, and developmental context. Antagonistic facets of grandiose narcissism were consistently linked to colder and more conflictual parent-child relationships, whereas agentic facets showed neutral or context-dependent associations. Vulnerable narcissism was more strongly associated with child maladjustment through mechanisms such as attachment insecurity, scapegoating, and maladaptive parenting practices. Overall, the findings underscore the importance of differentiating narcissism subtypes and identifying relational mediators to clarify pathways of risk and to inform targeted preventive and clinical interventions.

## Introduction and background

The parent-child relationship constitutes the cornerstone of children’s psycho-emotional development, shaping early expectations of interpersonal interactions, emotional regulation capacities, and the foundations of identity formation [1]. When this relationship is compromised by parental psychopathology, particularly personality disorders, the ability to respond consistently and sensitively to a child’s needs is disrupted [2]. Attachment theory posits that secure attachment develops when caregivers are consistently responsive and emotionally available, enabling children to regulate affect, explore their environment, and form stable internal working models of relationships [1]. In contrast, insecure attachment emerges in the context of inconsistent, rejecting, or emotionally dysregulated caregiving and is commonly associated with heightened emotional distress, maladaptive emotion regulation strategies, and increased vulnerability to internalizing and externalizing psychopathology across development [1,2].

Narcissistic personality disorder (NPD), defined by an excessive need for admiration, a lack of empathy, and a distorted self-perception, is particularly concerning in this context [3]. Conceptually, NPD comprises two distinct dimensions: grandiose narcissism and vulnerable narcissism [4,5]. Grandiose narcissism is associated with inflated self-image, dominance, entitlement, and a propensity to exploit others, often accompanied by emotional coldness and lack of reciprocity [6,7]. Parents with such traits may view their child as an extension of the self, reinforcing achievement and conformity to parental expectations while withholding affection when the child demonstrates independence or perceived weakness [6,7]. In contrast, vulnerable narcissism is marked by fragile self-esteem, emotional hypersensitivity, and heightened reactivity to perceived criticism [8]. Parents characterized by this profile frequently misinterpret their child's behavior, reacting with distress and expecting the child to satisfy their own need for reassurance. This interaction risks leading to "parentification," a situation where the child takes on a caregiving role for the emotionally unstable parent [9].

The present review conceptualizes NPD in accordance with the DSM-5, which characterizes NPD as a pervasive pattern of grandiosity (in fantasy or behavior), a need for admiration, and a lack of empathy [1,10]. However, contemporary research differentiates between grandiose and vulnerable subtypes, often operationalized through trait-based measures such as the Narcissistic Personality Inventory (NPI) and the Pathological Narcissism Inventory (PNI) [4]. The studies reviewed, spanning from 2015 to 2024, employed both DSM-aligned clinical assessments and dimensional self-report scales, reflecting a convergence between diagnostic and personality frameworks.

Although research has demonstrated that children of narcissistic parents are more likely to develop psychosocial difficulties such as low self-esteem, anxiety, and impaired interpersonal functioning across the lifespan [11,12], systematic exploration of these outcomes remains limited. Empirical studies suggest that exposure to parental narcissism fosters a developmental environment characterized by emotional inconsistency, conditional acceptance, and an absence of secure attachment [11]. Such conditions may result in disturbances of self-image, maladaptive emotion regulation, heightened vulnerability to affective disorders, and, in some cases, the intergenerational transmission of narcissistic or addictive behavioral patterns through internalization of dysfunctional relational models [12].

Recent developments in the study of personality psychopathology have reconceptualized NPD as a dimensional construct, characterized by maladaptive variations in self-esteem regulation, empathy, and interpersonal functioning [13,14]. Within the DSM-5 Alternative Model for Personality Disorders [1], narcissistic pathology is depicted as impairments in identity and self-direction, alongside challenges in empathy and intimacy. This framework is consistent with the Hierarchical Taxonomy of Psychopathology model, which positions narcissism within the antagonistic externalizing spectrum [15]. The integration of these perspectives facilitates a more nuanced comprehension of how grandiose and vulnerable narcissism differentially affect parenting behaviors and child development. Consequently, the present review synthesizes findings through this multidimensional lens, examining both clinical and subclinical manifestations of NPD within family dynamics.

This review aims to integrate existing research on the effects of parental NPD on the parent-child relationship and the psychological health of children. In this review, the parent-child relationship is conceptualized as the quality of emotional connection and interaction between parent and child, encompassing dimensions such as emotional availability, warmth, responsiveness, attachment security, communication patterns, and parenting behaviors, as assessed across the included studies. The review focuses on clarifying the processes by which parental narcissism affects parental actions, expectations, and the emotional interactions within the dyad, thereby shaping the child's developmental path. The review seeks to uncover critical pathways that could guide the development of preventive measures and therapeutic interventions.

## Review

Materials and methods

This systematic review was conducted in accordance with the Preferred Reporting Items for Systematic Reviews and Meta-Analyses (PRISMA) guidelines [16] and the methodological principles outlined for diagnostic accuracy reviews and followed the methodological framework for diagnostic accuracy reviews. The protocol was registered in the International Prospective Register of Systematic Reviews (PROSPERO) with ID number CRD420251139428.

Search Strategy

A comprehensive literature search was performed in four electronic databases: PubMed, PsycINFO, Scopus, and Google Scholar. The search covered the period from 1 January 2015 to 30 April 2024. Boolean operators were used to combine search terms, applying the following structure: “narcissistic personality disorder”, “parental narcissism”, “maternal narcissism”, “paternal narcissism”, “vulnerable narcissism”, “grandiose narcissism”, “attachment”, “parent-child relationship”, “child development”, and “child mental health. Filters were applied to restrict results to peer-reviewed, quantitative studies published in English. Reference lists of included articles and relevant reviews were hand-searched to identify additional eligible studies (Table 1).

**Table 1:** Electronic database search strategies

Database	Search string (exact query used)	Date of last search	Notes/filters applied
PubMed	(“narcissistic personality disorder”[Title/Abstract] OR “parental narcissism”[Title/Abstract] OR “maternal narcissism”[Title/Abstract] OR “paternal narcissism”[Title/Abstract]) AND (“parent–child relationship” OR “attachment” OR “child well-being” OR “child development”)	April 2024	Filters: english language; publication years 2015-2024; humans only
PsycINFO	(DE “Narcissism” OR DE “Personality Disorders”) AND (parent* OR maternal OR paternal) AND (child OR adolescent OR offspring)	April 2024	Filters: peer-reviewed journal articles; English language only
Scopus	TITLE-ABS-KEY (“narcissistic personality disorder” OR “narcissism”) AND TITLE-ABS-KEY (“parent*” OR “mother” OR “father”) AND TITLE-ABS-KEY (“child” OR “attachment” OR “development”)	April 2024	Filters: document type = article; language = english; year = 2015-2024
Google Scholar	“parental narcissism” AND (“child well-being” OR “attachment” OR “psychological adjustment”)	April 2024	First 200 results screened; duplicates removed manually

Inclusion and Exclusion Criteria

The inclusion criteria were (a) empirical, peer-reviewed quantitative studies including cross-sectional, longitudinal, and prospective cohort designs; (b) studies assessing parental NPD or narcissistic traits as the main exposure variable; (c) child or adolescent psychological, emotional, or attachment outcomes; and (d) publication in English between 2015 and 2024. The exclusion criteria were (a) qualitative studies, reviews, meta-analyses, case reports, or commentaries; (b) studies examining narcissism as an outcome variable rather than exposure; (c) papers where parental NPD was not explicitly measured; (d) studies published in languages other than English; and (e) duplicate or non-original publications.

Selection Process

All records retrieved from the databases were screened independently by two reviewers (E.O. and A.S.). Titles and abstracts were assessed for relevance, followed by full-text evaluation based on the predefined inclusion and exclusion criteria. Disagreements were resolved through discussion until consensus was achieved. Ultimately, eight studies met the inclusion criteria and were included in the review.

Quality and Risk of Bias Assessment

The methodological quality of included studies was assessed using the Newcastle-Ottawa Scale (NOS) [17], adapted for cross-sectional and longitudinal designs. Each study was scored on three domains: (a) selection of participants, (b) comparability of groups, and (c) outcome assessment. Scores ranged from 5 to 8 out of 9, indicating moderate to high quality. No study was excluded based on quality rating, but lower-scoring studies were given less interpretive weight in the synthesis.

During the review process, minor deviations from the original protocol were made to enhance methodological precision and align the inclusion criteria with the characteristics of the available literature. Specifically, (a) the inclusion criteria were expanded to encompass studies that assessed clinically relevant narcissistic traits using validated psychometric instruments (e.g., NPI, PNI, and Hypersensitive Narcissism Scale (HSNS)) rather than only those involving formal DSM-5 diagnoses of NPD, due to the scarcity of clinically diagnosed samples in the empirical literature; (b) the outcome scope was broadened beyond child psychological symptoms to include relational and attachment-related outcomes, reflecting recurring empirical themes across eligible studies; and (c) language restrictions were limited to English-language publications to ensure methodological consistency and feasibility.

Data Extraction and Synthesis

All records retrieved from the databases were exported to EndNote X9 (Clarivate Plc, Philadelphia, PA, USA) for duplicate removal. Two independent reviewers (E.O. and A.S.) conducted the data extraction. They classified the studies inductively according to the outcomes associated with parental NPD and their impact on children. Both reviewers independently extracted data from all included studies to ensure consistency and minimize bias. Each study was evaluated against the inclusion criteria, and disagreements were resolved through discussion or consultation with a third reviewer (E.V.) until consensus was achieved. The quality of each study was independently assessed by two reviewers (E.O. and A.S.), with any discrepancies resolved through discussion or consultation with a third reviewer (E.V.). No automation tools were used during the screening process. Extracted items included author, year, country, sample characteristics, study design, parental narcissism measure, child outcome(s), mediators/moderators, primary findings, and quality assessment score. The extracted data were synthesized narratively, with particular attention to distinguishing between grandiose and vulnerable forms of NPD and their differential associations with child outcomes. Findings were grouped thematically by type of narcissism, outcome domain (e.g., parent-child relationship quality, child internalizing and externalizing symptoms, maladjustment, maternal well-being), and tested mechanisms (e.g., parenting style, parental cognitions, attachment processes). This thematic synthesis identified both consistent patterns and divergent results across studies, highlighting the roles of mediating and moderating factors, including parental overvaluation, scapegoating, maternal perception of the child as difficult, and attachment security.

Results

**Figure 1:**
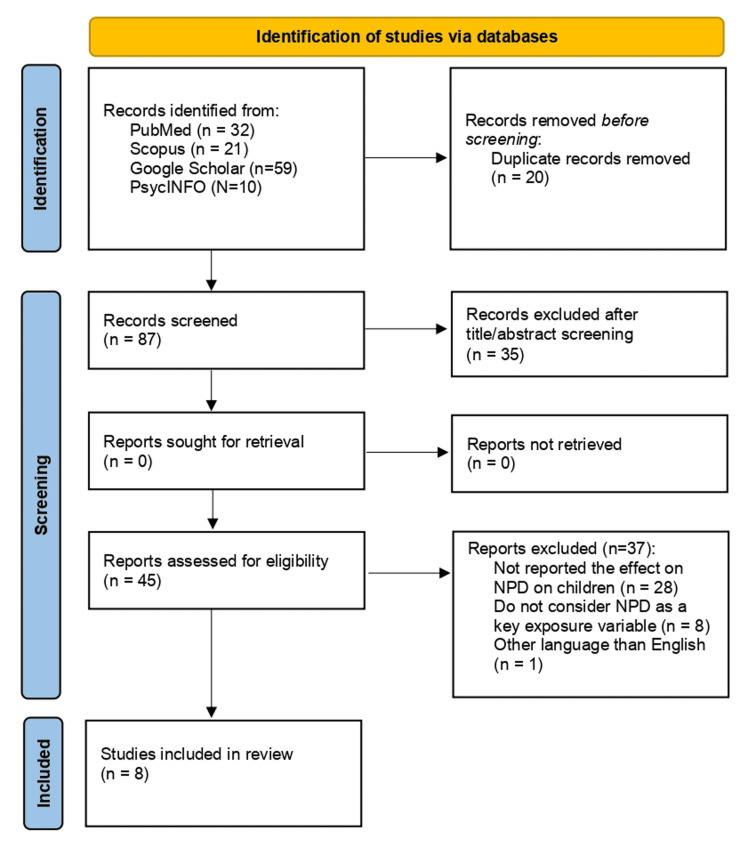
Flowchart of the study selection process NPD: narcissistic personality disorder

A total of eight studies met the eligibility criteria and were included in the review. The study selection process is presented in Figure 1.

A total of eight studies met the inclusion criteria and were included in the review. The characteristics of the included studies are summarized in Table 2.

**Table 2:** Characteristics of the included studies BDI: Beck Depression Inventory, STAI: State-Trait Anxiety Inventory, CBCL: Child Behavior Checklist, NPI: Narcissistic Personality Inventory, NPI-16: Narcissistic Personality Inventory-16 item version, NPI-40: Narcissistic Personality Inventory-40 item version, PNI: Pathological Narcissism Inventory, B-PNI: Brief Pathological Narcissism Inventory, SB-PNI: Super Brief Pathological Narcissism Inventory, HSNS: Hypersensitive Narcissism Scale, PHQ-9: Patient Health Questionnaire-9, ASQC: Attachment Style Questionnaire for Children, IRI-C: Interpersonal Reactivity Index for Children, PAQ: Personality Assessment Questionnaire, PANAS: Positive and Negative Affect Schedule, SWLS: Satisfaction With Life Scale, EPDS: Edinburgh Postnatal Depression Scale

Author (year, country)	Sample (N, parent type)	Child age	Study design	Narcissism measure (subtype)	Narcissism measure (for children)	Child outcome(s)	Reporter(s)	Mediators/moderators tested	Main findings	Quality assessment (NOS)
Jung and Schröder-Abé (2025, Germany)[13]	N = 1,743 parents; both mothers and fathers included	6-15 years old	Longitudinal (4 waves, 3 years, preregistered)	Grandiose narcissism (agentic vs. antagonistic subtypes)	Grandiose narcissism (agentic vs. antagonistic subtypes)	Parent-child relationship quality: intimacy, admiration, monitoring, conflict, negative communication	Both the parent self-report and the child report	None explicitly tested; direct associations and longitudinal prediction examined	Cross-sectional: antagonistic narcissism is negatively related to intimacy, admiration, warmth, and monitoring; positively to conflict and negative communication. Agentic narcissism is unrelated or weakly positive. Longitudinal: no evidence that narcissism predicted changes in relationship quality over time.	⭐ 8/9: very strong design, preregistered, representative national panel, multi-informant; limitation = attrition and small effect sizes over time.
Coppola et al. (2020, Italy) [5]	N = 519 children + parents (mothers = 494, fathers = 442)	9-11 years old	Cross-sectional, community-based school sample	Parental narcissism (NPI-16)	Child narcissism (Childhood Narcissism Scale)	Child narcissism, child self-esteem	Parents (self-report), children (self-report)	Parental overvaluation (mediator), parenting style (moderator)	Both mothers’ and fathers’ narcissism was positively associated with child narcissism. Fathers: effect partially mediated by overvaluation. Mothers: direct association. Positive maternal parenting predicted higher child self-esteem.	⭐ 6/9: large school-based sample, validated measures, mediation; single-informant on parenting, cross-sectional limits.
Vignando and Bizumic (2023, Australia) [18]	N = 504 emerging adults; retrospective reports on both parents	18-30 years old	Cross-sectional, online survey	HSNS (vulnerable), NPI-13 (grandiose), modified for parental rating	Anxiety (CUXOS), depression (CUDOS)	Anxiety, depression	Emerging adults (self-report, retrospective on parents)	Scapegoating (family scapegoating matrix) as mediator	Paternal grandiose narcissism directly predicted higher anxiety and depression. Maternal and paternal vulnerable narcissism predicted anxiety and depression indirectly via scapegoating. Maternal grandiose narcissism predicted outcomes indirectly via scapegoating. Scapegoating strongly predicted both anxiety and depression.	⭐ 5/9: large sample, validated tools, mediation tested; retrospective reporting, convenience sampling, cross-sectional.
Hewitt et al. (2024, Canada) [10]	N = 59 parent–child dyads; parents, mostly mothers)	9-16 years old (mage = 13.1)	Longitudinal, two waves, 1-year follow-up	SB-PNI - pathological narcissism (grandiose and vulnerable)	Child depression (PHQ-9), attachment (ASQC), perspective-taking (IRI-C)	Child depression: attachment perspective-taking	Parents (self-report narcissism), children (self-report outcomes)	Attachment anxiety and avoidance, affective and cognitive perspective-taking	Parental pathological narcissism predicted higher child depression after 1 year (controlling for baseline). Significant indirect effect via child attachment anxiety. No mediation via avoidance or perspective-taking. The model explained ~58% of the variance in depression.	⭐ 7/9: strong design, validated measures, multi-informant, mediation tested; small sample size and community recruitment lower representativeness.
Rawn et al. (2023, USA) [19]	N = 457 parents (59% mothers, 41% fathers)	6-18 years old	Cross-sectional, online survey (MTurk sample)	NPI-40; grandiosity, entitlement/exploitation (E/E), leadership/authority (L/A)	Child socio-emotional well-being: internalizing and externalizing symptoms (CBCL)	Child socio-emotional well-being	Parents (self-report on narcissism, parenting, child outcomes)	Positive parenting (warmth, autonomy support, democratic discipline); negative parenting (punitive, permissive discipline)	Grandiosity and E/E are linked to more negative and less positive parenting. Negative parenting mediated associations with higher child internalizing and externalizing. L/A is linked to more positive parenting but unrelated to child maladjustment.	⭐ 5/9: large sample, validated tools, SEM; but MTurk convenience sample, single-informant, cross-sectional.
Estlein et al. (2024, Israel) [8]	N = 252 mother–child dyads; mothers Mage = 40.8	7-12 years old (Mage = 9.0)	Cross-sectional, dyadic survey	B-PNI; grandiose and vulnerable	Child maladjustment (PAQ – child self-report)	Child maladjustment	Mothers (narcissism, parenting, perception of child); children (maladjustment)	Maternal perception of child as difficult; maternal acceptance/rejection parenting style	Vulnerable narcissism predicted higher child maladjustment, fully mediated by the perception of the child as difficult. Rejecting parenting was correlated with, but not a significant mediator of, the relationship. The model explained 26% of the variance.	⭐ 6/9: dyadic design, validated tools, mediation tested; limited generalizability (highly educated mothers), cross-sectional.
Dentale et al. (2015, Italy) [6]	N = 409 young adults, both parents also participated (mothers' mage ≈ 50 yrs; fathers' mage ≈ 54 yrs)	Mage = 22.9 years old	Cross-sectional, dyadic family study (parents + adult offspring)	NPI (parent self-report)	Depression (BDI), anxiety (STAI)	Depression, anxiety	Parents (NPI), children (PBI, BDI, STAI)	Parental rearing style (PBI: care, overprotection, putdown/shaming, favouritism)	Parental narcissism correlated with higher child depression and anxiety. Associations fully mediated by rearing style (affectionless control). Indirect effects are significant for both maternal and paternal narcissism; direct effects are nonsignificant.	⭐ 6/9: large sample, validated measures, mediation tested; limitation = retrospective recall bias, cross-sectional.
Talmon et al. (2021, Israel) [20]	N = 385 Israeli women; assessed during pregnancy (T1, M = 27.4 weeks) and 2 months postpartum (T2); mage ≈ 31	Not applicable	Longitudinal, pregnancy → postpartum (2 waves)	B-PNI (grandiose and vulnerable), maternal postpartum well-being (PANAS, SWLS, EPDS); bonding (Postpartum Bonding Questionnaire)	Not applicable	Maternal postpartum well-being, depression, and bonding	Mothers (self-report, both time points)	Body experience, maternal self-efficacy, prenatal attachment to fetus, postpartum bonding	Grandiose narcissism predicted better postpartum well-being and stronger mother–infant bonding indirectly via positive body experience, stronger prenatal attachment, and higher maternal self-efficacy.	⭐ 7/9: large sample, validated measures, longitudinal; self-report only and attrition bias reduce score.

Among the studies included, the majority were cross-sectional, with only two longitudinal [10,13]. The studies were conducted in diverse countries, including the United States [19] and Canada [10], Australia [18], Israel [8,20], Italy [5,6], and Germany [13]. All participants were assessed with standardized psychometric tools. Only two studies focused exclusively on mothers, whereas the rest examined parental samples including both fathers and mothers. Most studies investigated both grandiose and vulnerable forms of narcissism, while two examined grandiose narcissism specifically, and another two assessed narcissism more generally without subtype differentiation (Table 2).

Across eight studies spanning community and panel samples, evidence converges on the conclusion that parental narcissism is associated with poorer child and family outcomes, with effect size, pathway, and even direction varying systematically by narcissism subtype, facet, informant, and developmental context. In the analysis below, findings are systematically arranged by the specific type of narcissism and the relevant outcome domain, with a focus on mechanisms that have been empirically tested (Table 2).

Quality and Risk of Bias Assessment

The methodological quality and risk of bias of the included studies were assessed using the NOS adapted for cross-sectional and longitudinal designs. Overall, study quality ranged from moderate to high, with total NOS scores ranging from 5 to 8 out of 9. Most studies demonstrated adequate participant selection and outcome assessment. However, limitations were commonly observed in comparability domains, primarily due to cross-sectional designs, reliance on self-report measures, and limited control for potential confounding variables. No study was excluded based on quality assessment. Detailed quality ratings for each included study are presented in Table 3. While an overall quality rating is summarized in Table 2, a detailed breakdown of the risk-of-bias domains for each study is presented in Table 3.

**Table 3:** Quality and risk of bias assessment of included studies NOS: Newcastle-Ottawa Scale

Study (author, year)	Study design	Selection (0-4)	Comparability (0-2)	Outcome (0-3)	Total NOS score	Overall quality
Jung and Schröder-Abé (2025)	Longitudinal	4	2	2	8	High
Coppola et al. (2020)	Cross-sectional	3	1	2	6	Moderate
Vignando and Bizumic (2023)	Cross-sectional	3	1	1	5	Moderate
Hewitt et al. (2024)	Longitudinal	4	2	1	7	High
Rawn et al. (2023)	Cross-sectional	3	1	1	5	Moderate
Estlein et al. (2024)	Cross-sectional	3	1	2	6	Moderate
Dentale et al. (2015)	Cross-sectional	3	1	2	6	Moderate
Talmon et al. (2021)	Longitudinal	4	2	1	7	High

Grandiose Narcissism

Grandiose narcissism showed a differentiated profile across studies. In this context, antagonistic facets refer to traits characterized by entitlement, exploitativeness, hostility, and low empathy. In contrast, agentic or leadership facets reflect assertiveness, dominance, self-confidence, and a tendency toward socially assertive or leadership-oriented behaviors. When parsed into antagonistic versus agentic/leadership facets, antagonistic traits were consistently associated with colder, more conflictual parent-child relationships. In contrast, agentic facets were generally unrelated to, or showed only weak positive associations with, relational outcomes, such as parental monitoring, involvement, or warmth, particularly in cross-sectional analyses [13]. In models investigating parenting mechanisms, heightened levels of grandiosity and entitlement/exploitation are associated with increased negative parenting behaviors and reduced positive parenting practices. These traits serve as indirect pathways linking parental narcissism to children’s internalizing and externalizing difficulties, with internalizing difficulties referring to symptoms such as anxiety, depression, and emotional withdrawal, and externalizing difficulties encompassing behavioral problems such as aggression, oppositional behavior, and rule-breaking [5,19]. Notably, across studies examining child mental health and attachment outcomes, grandiose narcissism did not show consistent direct associations with children’s psychological symptoms or attachment security, with effects more often emerging indirectly through parenting behaviors or family-level processes rather than as direct effects [18].

Intergenerationally, fathers’ grandiose traits predicted children’s narcissism partly through parental overvaluation (defined as parents’ exaggerated and unrealistic positive appraisals of their child’s specialness, entitlement, and deservingness independent of actual performance), while mothers’ grandiose traits related more directly to child narcissism; children’s self-esteem, by contrast, tracked maternal positive parenting rather than parental narcissism, underscoring distinct developmental routes for narcissism versus self-esteem [5]. In emerging-adult retrospective data, perceived paternal grandiose narcissism had small direct associations with anxiety and depression. In contrast, perceived maternal grandiose effects were indirect via scapegoating dynamics within the family, defined as a dysfunctional family process in which one child is persistently blamed, devalued, or assigned responsibility for family tensions or parental distress, thereby increasing vulnerability to anxiety and depressive symptoms [18]. In the specific transition-to-motherhood context, grandiose traits predicted better short-term maternal well-being postpartum through more positive body experience, stronger prenatal attachment that carried forward into bonding, and higher maternal self-efficacy, suggesting situational advantages of agentic self-enhancement in early caregiving tasks [20].

Vulnerable (Pathological) Narcissism

Vulnerable features showed the most consistent adverse associations across studies and outcomes. In mother-child dyads, maternal vulnerable narcissism, rather than grandiose, predicted greater child maladjustment, fully explained by the mother’s perception of the child as “difficult,” while rejecting parenting did not add explanatory power once maternal perception was considered [8]. Among emerging adults, perceived maternal and paternal vulnerable narcissism related to higher anxiety and depression indirectly through scapegoating, highlighting a cognitive-relational mechanism by which vulnerable traits translate into offspring internalizing symptoms [18]. Longitudinally, higher parental pathological narcissism at baseline predicted increases in child depression one year later, with anxious attachment serving as the mediator and avoidant attachment and perspective-taking playing no explanatory role, providing rare temporal evidence for an attachment-based pathway from parental narcissism to child depressive symptoms [10]. In the perinatal context, vulnerable narcissism forecasts poorer maternal well-being via less positive body experience, lower self-efficacy, and more bonding difficulties, mapping a coherent chain from self-fragility to impaired early caregiving adaptation [20].

Recent longitudinal studies [18,21] have further illuminated the temporal associations between parental narcissism and child depressive symptoms, confirming attachment insecurity as a consistent mediator across developmental periods. These findings complement earlier cross-sectional research and reinforce the intergenerational nature of narcissistic vulnerability.

Parenting Climate, Cognitions, and Socialization Pathways

Two families of mechanisms recurred. First, parenting style/tactics: lower warmth and autonomy support and higher punitive/permissive discipline mediated links from antagonistic grandiose facets to child internalizing/externalizing problems [19]. Second, parental cognitions/attributions: overvaluation transmitted fathers’ narcissism to child narcissism [5], maternal perception of the child as difficult explained vulnerable-to-maladjustment links [8], scapegoating conveyed both maternal/paternal vulnerable-and maternal grandiose-effects to offspring anxiety/depression [18], and anxious attachment accounted for prospective effects of parental pathological narcissism on child depression [10]. Together, these findings suggest that how narcissistic parents see and use the child-overvalued extension, difficult foil, and scapegoat may be as consequential as overt tactics. That child insecurity in close relationships is a plausible downstream mediator (Table 2).

Outcome-Specific Patterns

For internalizing outcomes (depression/anxiety), evidence spans retrospective young-adult designs, parent-report models with parenting mediators, and a prospective dyadic study. Effects were typically indirect via scapegoating, rearing style, or attachment, with the clearest prospective pathway documented through child anxious attachment (Table 2). In the context of externalizing behaviors, the consistent finding was an indirect risk mediated by harsher or inconsistent parenting, which was associated with antagonistic grandiose traits [6]. Regarding global maladjustment, maternal vulnerable narcissism was found to predict child difficulties through maternal perception biases [8]. Regarding the quality of the parent-child relationship, antagonistic grandiosity co-occurred with reduced intimacy, admiration, and monitoring, alongside increased conflict and negative communication. However, it did not drive changes over time at the population level [13]. Finally, for maternal well-being and bonding postpartum, grandiose versus vulnerable traits showed opposite, mediation-rich profiles, indicating subtype-specific adaptation during early motherhood [20].

Consistencies, Contingencies, and Boundary Conditions

Three cross-study consistencies stand out. First, vulnerable narcissism is the more reliable correlate of adverse child outcomes across reporters and contexts, largely via perception-, scapegoating-, and attachment-based mechanisms (Table 2). Second, grandiose narcissism is facet-sensitive: antagonistic features track poorer relationship climate and parenting, whereas agentic/leadership features can coincide with more positive tactics or short-term maternal adjustment, helping explain mixed direct associations with child symptoms. Third, pathways are mostly indirect, emphasizing the leverage points of parenting behaviors, parental cognitions, and children’s attachment security rather than strong direct effects on symptoms. Contingencies include parent gender, with clearer direct risk linked to paternal grandiose traits in one retrospective study [18] and father-specific overvaluation pathways in intergenerational narcissism [5]. Additionally, informant effects are considered, as single-informant cross-sectional designs yield stronger concurrent associations than do multi-informant longitudinal panels focused on change (Table 2).

Discussion

This review highlighted important evidence for the impact of parental NPD on the quality of the relationship with the child and the child's well-being. The findings of this review highlight the complex and diverse impacts of parental NPD on both the quality of the parent-child relationship and the child's mental health, consistent with recent theoretical developments in the conceptualization of NPD. It is essential to distinguish between the grandiose and vulnerable forms of NPD, as each uniquely affects parental behavior and child developmental outcomes.

The vulnerable form of NPD was consistently identified as the most detrimental, being associated with reduced emotional availability, distorted perceptions of the child’s behavior, heightened emotional reactivity, and difficulties in regulating parental demands [22]. Such characteristics frequently destabilize attachment relationships, promote role reversal phenomena such as parentification, and increase the risk of depressive and anxiety symptoms in children and adolescents [23]. These findings align with broader evidence suggesting that parental NPD exerts indirect effects through mechanisms including emotional dysregulation, deficits in empathy, and insecure attachment [3,24].

Parental narcissism significantly disrupts secure attachment formation, which is critical for healthy emotional regulation and mental health outcomes in children [21]. According to attachment theory, secure attachment arises from responsive caregiving, but narcissistic parents often prioritize their own needs over those of their children, leading to insecure attachment styles [23]. Hewitt et al. [10] highlight that parental pathological narcissism is positively associated with later child depression, anxious and avoidant attachment, and perspective taking. Moreover, Dentale et al. [6] demonstrate that parental narcissism correlates with children's mental vulnerability, suggesting that maladaptive parenting styles mediate this relationship. Collectively, this study highlights the detrimental effects of parental narcissism on children's secure attachment and subsequent emotional regulation, emphasizing its long-term implications.

In contrast, the grandiose form of NPD did not show consistent direct associations with child mental health or attachment outcomes across studies, with effects often emerging indirectly or varying depending on contextual and relational factors [22]. While it is often associated with authoritarian, antagonistic, or emotionally distant parental practices [9], the current evidence base remains insufficient to establish a direct causal link with internalizing symptoms such as depression or anxiety in children. Recent studies have illuminated a fascinating link between the grandiose dimension of personality and heightened bonding levels during the postpartum period [25,26]. This relationship appears context-dependent, suggesting that certain situations may amplify the positive effects of grandiosity on parent-child bonding. The temporary feelings of self-affirmation and control that accompany the parental role appear to be crucial in this dynamic, potentially enhancing the parent's sense of importance and capability. This can be understood in light of the behavioral profile of grandiose narcissism, which includes highly performative parenting behaviors aimed at gaining admiration and compliance from children, often producing superficially adaptive outcomes [9]. In this context, the absence of a direct association between maternal grandiose narcissism and child maladjustment may be explained by the possibility that such parental behaviors are perceived as socially advantageous, thereby fostering temporary or situationally beneficial outcomes for children’s social and emotional development [11,26-30].

One study [20] examined the association between grandiose traits and postpartum bonding, suggesting that higher levels of parental grandiosity are linked to poorer quality of early parent-child attachment, primarily through reduced emotional sensitivity and increased self-focused caregiving behaviors. Further research is warranted to determine whether similar patterns are observed across broader parental populations and longitudinal designs.

By synthesizing a decade of research, including recent multi-informant and longitudinal data, this review builds upon previous analyses and offers an updated theoretical consolidation of the field. These contributions enhance the empirical foundation of the proposed theoretical model, situating parental narcissism within broader frameworks of attachment, emotional regulation, and the intergenerational transmission of psychopathology. This synthesis advances existing literature by systematically differentiating between the vulnerable and grandiose aspects of parental NPD, thereby revealing distinct developmental pathways for child maladjustment. By identifying mediating processes such as scapegoating, overvaluation, and attachment insecurity, this review pinpoints specific targets for clinical intervention. These findings extend beyond individual studies by integrating multi-informant, cross-national evidence into a cohesive conceptual model that links parental narcissism to child mental health. Taken together, the findings of this review have several important practical implications for clinical practice, prevention, and family-focused interventions.

The findings of this review carry several important practical implications for clinical practice, prevention, and family-focused interventions. First, the differentiated effects of parental narcissism, particularly the distinction between antagonistic and agentic facets of grandiose narcissism, highlight the need for nuanced assessment approaches in both clinical and community settings. Rather than treating parental narcissism as a uniform risk factor, clinicians should attend to specific trait configurations, as antagonistic traits appear more strongly associated with relational coldness, conflict, and maladaptive parenting patterns. In contrast, agentic traits may confer context-dependent advantages in specific caregiving periods, such as the transition to parenthood.

Second, the consistent identification of indirect pathways linking parental narcissism to child outcomes underscores the central role of parenting behaviors and family dynamics as intervention targets. Mechanisms such as negative parenting practices, parental overvaluation, scapegoating, and attachment-related processes suggest that interventions aimed at enhancing parental sensitivity, emotional attunement, and reflective functioning may mitigate the adverse impact of narcissistic traits on children, even in the absence of change at the personality trait level. This is particularly relevant given the relative stability of narcissistic traits across adulthood.

Third, the evidence that grandiose narcissism does not consistently exert direct effects on child mental health or attachment outcomes but instead operates through contextual and relational mechanisms supports the value of early, family-centered preventive interventions. Programs focusing on parenting support, attachment-based guidance, and emotion regulation skills, especially during sensitive developmental windows such as early childhood and the perinatal period, may buffer children from potential relational risks associated with parental narcissism.

Importantly, the findings related to the postpartum period suggest that not all manifestations of grandiose narcissism are uniformly maladaptive. In specific contexts, agentic self-enhancement may be associated with improved maternal well-being and bonding through increased self-efficacy and positive body experience. This highlights the importance of avoiding overly pathologizing interpretations and instead adopting a strengths-informed, context-sensitive approach when working with parents displaying narcissistic traits.

Finally, these results have implications for professional training and policy development. Mental health professionals working with families, educators, and perinatal care providers may benefit from training that enhances awareness of how narcissistic traits manifest in parenting contexts and how these traits interact with developmental and situational factors. Incorporating screening for maladaptive parenting patterns and relational risk markers into routine child and family services could facilitate early identification and targeted support for at-risk dyads.

While this review provides valuable insights, several limitations should be noted. The small number of included studies, heterogeneity of psychometric tools, and absence of large-scale prospective designs limit generalizability. Reliance on self-report measures may have introduced bias, and restricting studies to English-language publications from 2015 to 2024 may have excluded relevant research. Citation tracking was not performed, which may have resulted in missing additional studies. It is also worth noting that all included studies were conducted in high-income countries. This may accurately reflect the current state of the literature, or it may be influenced by the search strategy, the restriction to English-language publications, or the omission of gray literature, which could limit the review's comprehensiveness.

Nonetheless, this review represents one of the initial efforts to systematically synthesize recent empirical evidence on parental NPD and its impact on children. Future research should aim to systematically distinguish between grandiose and vulnerable NPD subtypes, incorporate prospective longitudinal data, and explore both maternal and paternal influences across diverse sociocultural contexts. Furthermore, investigating protective factors, such as secure attachment to alternative caregivers or the availability of psychosocial support, would offer crucial insights into resilience pathways for children exposed to narcissistic parenting.

## Conclusions

This review demonstrates that parental NPD, particularly in its vulnerable manifestation, significantly compromises parent-child relationships and child emotional well-being. Vulnerable narcissism is consistently associated with insecure attachment, role reversal, and increased anxiety and depression risk. Conversely, grandiose narcissism, while linked to antagonistic and authoritarian parenting, exhibits less consistent direct effects on children. In certain contexts, grandiose traits may temporarily align with adaptive behaviors, illustrating subtype-specific influences.

The evidence highlights the need for early identification of narcissistic traits and development of interventions aimed at enhancing emotional availability, restoring appropriate roles, and strengthening attachment security. Further research using longitudinal designs, diverse samples, and multi-informant approaches is essential to refine understanding and guide clinical strategies for families affected by parental NPD.

These findings underscore the importance of distinguishing between NPD forms in research and clinical settings. By identifying specific relational pathways, interventions can better address unique challenges posed by vulnerable versus grandiose narcissism. As a preliminary synthesis of emerging evidence, this review should be viewed as an exploratory effort that identifies consistent patterns and theoretical pathways but cannot establish causal mechanisms. Future systematic and longitudinal research is needed to confirm these relationships and to clarify developmental trajectories.

## References

[1] American Psychiatric Association (2013). Diagnostic and Statistical Manual of Mental Disorders, Fifth Edition.

[2] Baskin-Sommers A, Krusemark E, Ronningstam E (2014). Empathy in narcissistic personality disorder: from clinical and empirical perspectives. Personal Disord.

[3] Bloxsom CA, Firth JK, Kibowski F, Egan V, Sumich AL, Heym N (2021). Dark shadow of the self: how the dark triad and empathy impact parental and intimate adult attachment relationships in women. Forensic Sci Int Mind Law.

[4] Cain NM, Pincus AL, Ansell EB (2008). Narcissism at the crossroads: phenotypic description of pathological narcissism across clinical theory, social/personality psychology, and psychiatric diagnosis. Clin Psychol Rev.

[5] Coppola G, Musso P, Buonanno C (2020). The apple of daddy’s eye: parental overvaluation links the narcissistic traits of father and child. Int J Environ Res Public Health.

[6] Dentale F, Verrastro V, Petruccelli I, Diotaiuti P, Petruccelli F, Cappelli L, San Martini P (2015). Relationship between parental narcissism and children’s mental vulnerability: mediation role of rearing style. Int J Psychol Psychol Ther.

[7] di Giacomo E, Andreini E, Lorusso O, Clerici M (2023). The dark side of empathy in narcissistic personality disorder. Front Psychiatry.

[8] Estlein R, Gewirtz-Meydan A, Finzi-Dottan R (2024). Maternal narcissism and child maladjustment: a dyadic study. Current Psychology.

[9] Hart CM, Bush-Evans RD, Hepper EG, Hickman HM (2017). The children of narcissus: Insights into narcissists' parenting styles. Pers Individ Differ.

[10] Hewitt JM, Kealy D, Hewitt PL (2024). Parental pathological narcissism and child depression: the indirect effects of child attachment and perspective taking. Curr Psychol.

[11] Carlson EN (2012). Honestly arrogant or simply misunderstood? Narcissists’ awareness of their narcissism. Self Identity.

[12] Jabeen F, Gerritsen C, Treur J (2021). Healing the next generation: an adaptive agent model for the effects of parental narcissism. Brain Inform.

[13] Jung J, Schröder-Abé M (2025). The cross-sectional and longitudinal association of grandiose narcissism with the quality of the parent-child relationship. Collabra: Psychol.

[14] Krueger RF, Hobbs KA, Conway CC (2021). Validity and utility of hierarchical taxonomy of psychopathology (HiTOP): II. externalizing SuperSpectrum. World Psychiatry.

[15] Levy KN, Meehan KB, Cain NM, Ellison WD (2013). Narcissism in the DSM. Understanding and Treating Pathological Narcissism.

[16] Page MJ, McKenzie JE, Bossuyt PM (2021). The PRISMA 2020 statement: an updated guideline for reporting systematic reviews. BMJ.

[17] Wells G.A., Shea B., O’Connell D. (2000). The Newcastle-Ottawa Scale (NOS) for assessing the quality of nonrandomised studies in meta-analyses. https://ohri.ca/en/who-we-are/core-facilities-and-platforms/ottawa-methods-centre/newcastle-ottawa-scale.

[18] Vignando M, Bizumic B (2023). Parental narcissism leads to anxiety and depression in children via scapegoating. J Psychol.

[19] Rawn KP, Keller PS, Widiger TA (2025). Parent grandiose narcissism and child socio-emotional well being: the role of parenting. Psychol Rep.

[20] Talmon A, Finzi-Dottan R, Ginzburg K (2021). "I will love you (me) forever"-A longitudinal study of narcissism and emotional adjustment during the transition to motherhood. Personal Disord.

[21] Pincus AL, Lukowitsky MR (2010). Pathological narcissism and narcissistic personality disorder. Annu Rev Clin Psychol.

[22] Weinberg I, Ronningstam E (2022). Narcissistic personality disorder: progress in understanding and treatment. Focus (Am Psychiatr Publ).

[23] Saladino V, Cuzzocrea F, Calaresi D, Gullo J, Verrastro V (2024). Attachment styles, vulnerable narcissism, emotion dysregulation and perceived social support: a mediation model. Soc Sci.

[24] Zhang Y, Zhang J, Wang Y (2024). The relationship between attachment insecurity and pathological narcissism: a three-level meta-analysis. J Fam Theory Rev.

[25] Neda-Stepan O, Giurgi-Oncu C, Sălcudean A, Bernad E, Bernad BC, Enătescu VR (2024). The influence of personality traits on postpartum depression: a systematic review based on the neo-FFI scale. Diseases.

[26] Määttä M, Uusiautti S (2020). ‘My life felt like a cage without an exit’ - narratives of childhood under the abuse of a narcissistic mother. Early Child Dev Care.

[27] Rogoza R, Cieciuch J, Strus W (2022). Vulnerable isolation and enmity concept: disentangling the blue and dark face of vulnerable narcissism. J Res Pers.

[28] Sheffield Morris A, Cui L, Jespersen JE, Criss MM, Cosgrove KT (2022). Parenting and children’s social and emotional development: emotion socialization across childhood and adolescence. The Cambridge Handbook of Parenting.

[29] Zajenkowski M, Maciantowicz O, Szymaniak K, Urban P (2018). Vulnerable and grandiose narcissism are differentially associated with ability and trait emotional intelligence. Front Psychol.

[30] Zajenkowski M, Rogoza R, Maciantowicz O, Witowska J, Jonason PK (2021). Narcissus locked in the past: vulnerable narcissism and the negative views of the past. J Res Pers.

